# Symptoms of Depression and Anxiety After COVID-19 Despite Systematic Telemedical Care: Results From the Prospective COVID-SMART Study

**DOI:** 10.1155/da/9989990

**Published:** 2025-04-13

**Authors:** Aenne S. von Falkenhausen, Antonia Gail, Scott Geipel, Clemens Scherer, Sven Stockhausen, Lauren E. Sams, Finn Becker, Steffen Massberg, Stefan Kääb, Moritz F. Sinner

**Affiliations:** ^1^Department of Medicine I, LMU University Hospital, LMU Munich, Munich, Germany; ^2^German Centre for Cardiovascular Research (DZHK), Munich Heart Alliance, Munich, Germany

**Keywords:** anxiety, COVID-19, depression, Long-COVID, telemedical care

## Abstract

**Background:** Long-COVID has attracted increased attention with rising numbers of affected patients and high individual symptom burden. Prior studies have described its prevalence, course of disease, and severity. Yet, the influence of intensive care, including telemedical support for patients at risk for a severe course of the initial COVID-19 disease, on the occurrence of Long-COVID and its associated symptoms is studied to a lesser extent.

**Methods:** Here, we report the long-term results of the COVID-SMART study, which randomized at-risk COVID-19 patients to either smartwatch-based monitoring with telemedical support or standard care. We investigate Long-COVID symptoms, including symptoms of depression and anxiety after 12 months.

**Findings:** Between October 2020 and May 2022, we enrolled 607 patients in the COVID-SMART study. Complete 12-month follow-up was available for 573 patients, with 288 patients randomized to the intervention group and 285 to the control group. Overall, 234 participants (40.8%) reported COVID-related symptoms, with a high prevalence of symptoms of depression (209 participants, 36.5%) and anxiety (232 participants, 40.5%). However, telemedical support did not reduce these symptoms at follow-up. Multivariable regression analysis identified sex, active smoking, and pre-existing asthma as significant predictors of both outcomes.

**Interpretation:** COVID-SMART is the first prospective, randomized clinical trial to systematically assess the impact of telemedical care on the development of Long-COVID associated risk of depression and anxiety. We identify in part modifiable risk factors for these outcomes. However, telemedical support does not appear to be beneficial in reducing symptoms of anxiety and depression and should hence be focused to the acute infection phase.

**Trial Registration:** ClinicalTrials.gov identifier: NCT04471636

## 1. Introduction

With the emergence of COVID-19, it has become increasingly evident that both physical and psychological symptoms may persist beyond the acute disease period [1]. This has led to the introduction of “Long-COVID” as the cumulative characterization of the frequent phenomenon of persisting symptoms lasting from 4 weeks to 3 months or longer after the acute COVID-19 infection [2, 3]. Although the definition of Long-COVID varies, it encompasses multiple different health constraints, including ongoing respiratory and cardiac limitations and, most importantly, a wide range of psychological disturbances like ongoing depression and anxiety, fatigue, or sleep disturbances [4, 5].

Observational, population-based studies have concluded that Long-COVID is indeed common, with a wide prevalence ranging from 2.3% to 76% [2, 6–10], leading to significant reductions in personal well-being and both interpersonal and occupational functionality [11]. Focusing on depression and anxiety, it is assumed that up to 23% of patients report associated symptoms as part of Long-COVID [12–14].

Despite varying evidence in disease prevalence, some predictors for Long-COVID emerged, including female sex, older age, obesity, asthma, poor general or prepandemic health, and poor sociodemographic factors [6, 15]. Focusing on depression and anxiety after COVID-19, risk factors are similar and include female sex, lower socioeconomic status, and greater COVID-related stress exposure [16–19].

However, little is known about the effect of COVID-19-related therapeutic measures on the incidence of these Long-COVID symptoms. Particularly, nondrug-based measures like intensive telemedical support may influence the prevalence of depression and anxiety as part of Long-COVID. Telemedicine has already demonstrated its relevance in various areas of medicine; examples include the telemedical care of patients with chronic heart failure to help reduce hospitalizations and improve patients' quality of life [20]. Other important areas also include the App-based telemedical care of patients with symptoms of depression and anxiety, which has entered routine care [21, 22].

In contrast, scientific data on telemedical care for patients with Long-COVID are lacking. Here, telemedicine could play likewise an important role, especially as patients may perceive close telemedical support as an additional source of reassurance for the potential reduction of anxiety-related symptoms.

Here, we have analyzed data from the prospective, randomized COVID-SMART study to (1) systematically assess the prevalence of symptoms of depression and anxiety in a well-characterized, prospective cohort of outpatient COVID-19 patients in domestic isolation, to (2) identify predictors of elevated risk for depression and anxiety, and to (3) inform if intensive telemedical care may reduce symptoms of depression and anxiety during follow-up.

## 2. Methods

COVID-SMART is an investigator-initiated, prospective, randomized, open-label, controlled clinical trial that included patients with acute COVID-19 treated in domestic isolation. All patients had risk factors for an adverse course of the disease. The trial was conducted at the LMU University Hospital, LMU Munich, Germany. Study details have been reported before [23].

In brief, for inclusion into COVID-SMART, patients were ≥18 years of age, presented with a PCR-confirmed active COVID-19 infection within the preceding 7 days, qualified for outpatient medical care, and presented with at least one risk factor for an adverse course of the disease. Risk factors included treated or untreated arterial hypertension; active smoking of ≥ five cigarettes per day; body mass index ≥30 kg/m^2^; atrial fibrillation; diabetes mellitus; systolic or diastolic heart failure; coronary artery disease status post percutaneous coronary intervention or coronary artery bypass graft surgery. Participants had to have a smartphone with sufficient connectivity.

Participants were then 1:1 randomized into a control group receiving standard care and an intervention group that was additionally equipped with a smartwatch capable of measuring heart rate, oxygen saturation, and electrocardiogram recording. Furthermore, intervention group patients had access to a 24/7 hotline staffed with experienced physicians who could be contacted in case of pathologic smartwatch measurements, progressive COVID-related symptoms, or general inquiries. Randomization was computer-based, using the WolframAlpha randomization function in simple blocks of 50 participants (Wolfram Research, Wolfram Alpha LLC, United States). No stratification of confounding factors was performed.

In the intervention group, telemedical care was provided for 30 days. Telephone-based follow-up was conducted at 30 days and 12 months after inclusion.

### 2.1. Long-COVID-Related Outcomes

To assess physical and psychological impediments, we performed a detailed inquiry about the individual health status 12 months after the index COVID-19 infection. The follow-up included details about pre-existing and newly developed conditions, medication, hospitalizations, and vaccinations. Psychological impediments were assessed according to the S1 guideline for the diagnosis of Long-COVID, screening for symptoms of depression, anxiety, fatigue, posttraumatic stress disorder, compulsion, and somatization disorders [24]. This included screening questions for depression (Have you often felt down, sad, depressed, or hopeless in the last month? In the last month, have you had significantly less desire and pleasure in doing things that you normally enjoy?) and anxiety (Have you ever had an anxiety attack where you were suddenly overcome by fear, anxiety, and restlessness? Do you sometimes have unfounded fears, e.g., on public transport, in public places, of particular situations, objects or animals? Have you felt anxious, tense, or full of fearful worry in the last month or more?). The screening was considered positive if at least one question was answered affirmatively.

### 2.2. Statistical Analysis

We present discrete data as absolute and relative frequencies and continuous data as means±standard deviation or medians and interquartile ranges as appropriate. By randomization group, we calculated Fisher's exact tests to compare the prevalence of anxiety and depression. In the overall cohort, we identified predictors of the outcome of depression and anxiety during follow-up by fitting univariable logistic regression models. Significant predictors by univariable analysis for both depression and anxiety were included in multivariable-adjusted logistic regression models. All analyses were performed by R (version 3.6.1, The R Foundation for Statistical Computing, Vienna, Austria). Two-sided *p*-values < 0.05 were considered significant.

## 3. Results

**Figure 1:**
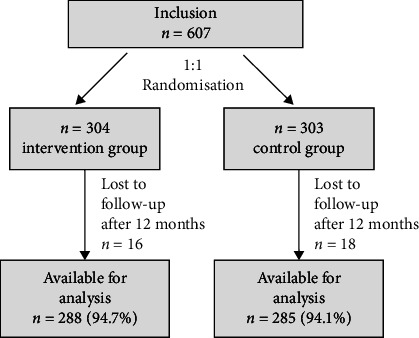
Study flow.

From October 2020 to May 2022, 607 patients with acute COVID-19 who were suitable for outpatient care participated in COVID-SMART. Of those, 304 were randomized into the intervention group and 303 into the control group [23]. For the current analysis, we included all 573 participants who completed our follow-up investigation 12 months after enrollment (*n* = 288 [94.7%] in the intervention group and *n* = 285 [94.1%] in the control group, Figure 1).

### 3.1. Baseline Characteristics

Patients had a mean age of 46.8 ± 13.3 years and were slightly predominantly male (54.6%) (Table 1). According to our inclusion criteria, all patients presented with a profound cardiovascular risk profile, particularly reflected by a high prevalence of atrial hypertension (47.6%), active smoking (28.6%), and diabetes mellitus (11.5%). Relevant concomitant diseases like asthma or autoimmune diseases were frequent (14.3% and 12.2%, respectively).

### 3.2. Prevalence of Long-COVID Symptoms at Follow-up

A large proportion of patients (*n* = 234, 40.8%) reported persisting symptoms 12 months after the index COVID-19 infection. Moderate or severe symptoms were reported by 64 participants (11.2%). In total, 147 participants (25.7%) experienced disabling symptoms at the time of follow-up. Reported symptoms encompassed a large spectrum, ranging from physical impairments, including skin changes (17.8%), cough (8.2%), dyspnea (13.4%), and palpitations (8.4%) to psychological problems like impaired concentration (12.9%), sleep-associated problems (12.4%), or perceived impaired memory (10.8%, Table 2).

### 3.3. Prevalence of Depression and Anxiety at 12 Months Follow-up

Twelve months after the index COVID-19 infection, 209 participants (36.5%) were screened positive for a high risk of depression. Participants at risk for depression were more likely female (*n* = 121 [57.9%] vs. *n* = 139 [38.2%], *p*  < 0.001), active smokers (*n* = 72 [34.4%] vs. *n* = 92 [25.3%], *p*=0.020), and suffered more frequently from pre-existing asthma (*n* = 39 [18.7%] vs. *n* = 43 [11.8%], *p*=0.025) and less frequently from hypertension (*n* = 87 [41.6%] v. *n* = 186 [51.1%], *p*=0.027, Table 1).

Similarly, 232 participants (40.5%) exhibited a high risk of anxiety disorder. Those participants were significantly younger (44.4 ± 12.9 vs. 48.4 ± 13.4 years, *p*  < 0.001), more likely female (*n* = 130 [56.0%] vs. *n* = 130 [38.1%], *p*=0.001) and actively smoking (*n* = 80 [34.5%] vs. 84 [24.6%], *p*=0.011). Patients at risk for anxiety had a higher prevalence of asthma (*n* = 47 [20.3%] vs. *n* = 35 [10.3%], *p*  < 0.001), autoimmune disease (*n* = 40 [17.2%] vs. *n* = 30 [8.8%], *p*=0.003) and liver disease (*n* = 23 [9.9%] vs. *n* = 18 [5.3%], *p*=0.038) but less frequently suffered from diabetes (*n* = 17 [7.3%] vs. *n* = 49 [14.4%], *p*=0.011) or atrial fibrillation (*n* = 5 [2.2%] vs. *n* = 24 [7.0%], *p*=0.012, Table 1).

**Figure 2:**
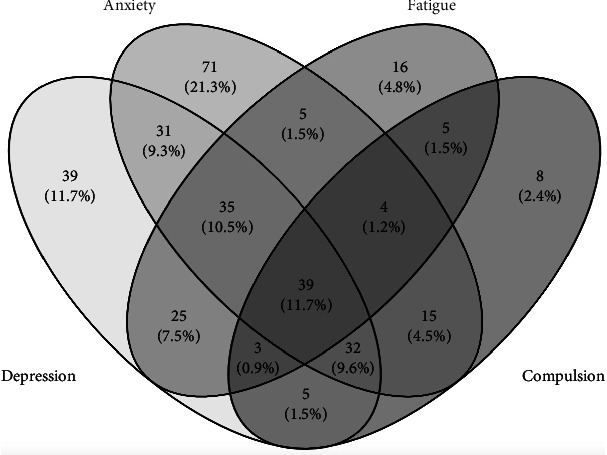
Venn diagram. The diagram shows an overlap between the presence of depression and anxiety symptoms with other psychosomatic or psychiatric symptoms like fatigue or compulsion.

There was a wide overlap between participants presenting with symptoms of depression and anxiety as well as other psychiatric and psychosomatic disorders like fatigue and compulsions (Figure 2).

### 3.4. Effect of Telemedical Support on Depression and Anxiety After COVID-19

Overall, 288 participants of our cohort received intensive telemedical care via smartwatch measurements and access to a 24/7 medical hotline support by an experienced medical team during their index COVID-19 home quarantine. Compared to the control group, we did not observe significant differences in Long-COVID-related symptoms or symptom severity (Table 3). When screening for an elevated risk of depression and anxiety, both groups exhibited similar prevalences of depression- (35.4% vs. 37.5%, *p*=0.604) and anxiety-related symptoms (40.3% vs. 40.7%, *p*=0.932). In a sensitivity analysis we found no relevant differences in the baseline characteristics of the cohort and those participants who were lost to follow-up. Interestingly, a smaller proportion of participants in the intervention group did not return to work 12 months after enrollment (6.9% vs 9.1%, *p*=0.04).

### 3.5. Predictors of Risk for Depression and Anxiety After COVID-19 in Outpatients

We included all baseline characteristics that differed significantly in both cohorts screened positive for depression and anxiety into a multivariable-adjusted model. Female sex, smoking status, and pre-existing asthma remained significantly associated with the presence of symptoms of depression and anxiety symptoms 12 months after the index COVID-19 infection. Female sex showed the strongest association with both conditions (odds ratio of females for depression (2.18 [1.54–3.12], *p*  < 0.001) and for anxiety-related symptoms (2.01 [1.42–2.84], *p*  < 0.001) (Table 4).

## 4. Discussion

We present results from the large, prospective, randomized COVID-SMART study that included patients at risk for severe COVID-19 who were managed as outpatients [23]. After 12 months of follow-up, we confirm that the prevalence of symptoms of depression and anxiety as part of Long-COVID is high, whereas telemedical support does not modify the risk of depression and anxiety, respectively, we report female sex, active smoking, and pre-existing asthma as significant predictors of both outcomes.

Prior studies have documented the high prevalence of Long-COVID symptoms in up to 68% at 6 months and 49% at 12 months after the initial COVID-19 infection [2]. Most importantly, symptoms of anxiety and depression significantly increased during the pandemic when compared to the time before the pandemic [25]. However, challenges remain when interpreting and comparing these previous results. First, clear definitions of the complex disease of Long-COVID are missing, and definitions vary between studies. This includes a lack of consensus on which symptoms of the complex spectrum of physical and psychological impediments can reliably be associated with Long-COVID and what timeframe should be used to classify symptoms as potentially disease-relevant [3, 26]. Second, most studies relied on retrospective data sets. Only a few studies were performed in a prospective way yielding higher data quality [27, 28]. Third, most studies did not focus on COVID-19 patients at risk for an adverse course of the disease, who were still managed as outpatients. Instead, previous studies commonly differentiated between hospitalized and nonhospitalized patients [29]. Our study, in turn, represents one of the first prospective, randomized, controlled clinical trials with high data quality and sufficiently complete follow-up information to systematically assess symptoms of depression and anxiety 12 months after their index COVID-19 infection. With 36.5% of participants reporting symptoms of depression and 40.5% reporting symptoms of anxiety, our results are well in line with previous reports and underline the high disease burden after COVID-19 infection.

Several risk factors for depression and anxiety as part of Long-COVID have been discussed, with data varying substantially. Female sex, however, has previously been identified as a relevant risk factor for later Long-COVID onset [30]. Our results highlight that the female sex is the most significant predictor for symptoms of anxiety and depression in a multivariable-adjusted analysis. The underlying pathophysiology is incompletely understood. A higher inflammation status with more relevant IgG antibody production and a different hormonal status in the early phase of the index COVID-19 disease have been considered as possible reasons for the higher prevalence of Long-COVID in females [31, 32]. However, these hypotheses require further endorsement. Some also postulate that women report symptoms of depression and anxiety more frequently than males and are more prone to depression and anxiety in general [33]. Nevertheless, even if a fundamental difference in the perception of disease relevance and severity cannot be fully ruled out, the association of female sex with the occurrence of Long-COVID remains highly relevant as women might need closer supervision during the COVID-19 recovery period to enable early identification of patients at risk and the initiation of treatment.

Previous analyses have reported that active smoking may represent a relevant risk factor for Long-COVID, as it was associated with a higher prevalence of tachycardia during follow-up [34]. Additionally, low socioeconomic status has repeatedly emerged as a potential risk factor and might also be correlated with higher tobacco use [35, 36]. In our analysis, we confirm that active smoking is significantly associated with an increased risk of depression and anxiety after COVID-19. However, future analyses are needed to elucidate if nicotine abuse can cause Long-COVID symptoms or if actively smoking individuals merely mark a socioeconomic subgroup that is more susceptible to depression and anxiety in general.

The correlation of asthma as a relevant risk factor for Long-COVID and depression has also been reported controversially. It has been clinically evident that patients with asthma are at increased risk for a severe course of COVID-19, including the consequence of a higher burden of symptoms. This more severe acute disease may thus potentially result in an increased risk for Long-COVID [37]. It has indeed been shown that asthma patients retain symptoms of dyspnea and worse asthma control after a COVID-19 infection [38, 39]. However, a correlation to an increased risk for depression and anxiety as part of Long-COVID has been missing so far. Our results, therefore, identify asthma as a potential risk factor for depression and anxiety as part of Long-COVID. Our finding is in line with data suggesting a correlation between asthma and fatigue [40].

Healthcare providers and future research should consider addressing the risk factors identified in our study. Gender-specific supervision during the COVID-19 recovery period and early initiation of psychological support might be beneficial. Similarly, additional health modification programs like smoking cessation programs might reduce the risk of depression and anxiety. For patients with asthma, optimizing disease control and providing tailored support may help mitigate the risk of long-term psychological consequences.

Importantly, it may be hypothesized that telemedical support could reduce symptoms of depression and anxiety by facilitating more personal contact and immediate medical attention if necessary. However, our study could not demonstrate such an effect. This neutral result may be partly explained by insufficient power due to our sample size. Given the observed effect sizes, a post hoc power analysis revealed that we achieved only 18.1% power for depression and 5.4% power for anxiety. Achieving 80% power to detect differences in the effect of such low magnitude would have required sample sizes of 8245 and 236,422 participants, respectively. However, the effort and burden to establish and maintain a 24/7 telemedical support system renders it questionable whether a significantly larger study is justifiable, considering our neutral result in almost 600 studied individuals. To our knowledge, this is the first study assessing the impact of telemedicine during the acute infectious course of COVID on long-term outcomes as part of Long-COVID. Despite our neutral result, these data are still relevant, as results from prospective, randomized studies are scarce in the context of COVID-19 and Long-COVID care, despite the very high number of affected individuals.

Some limitations need to be discussed. We report results from a single-center study performed in the region of Munich, Germany. Transferability to other geographic regions and healthcare settings may be limited. The assessment of depression and anxiety risk was performed by telephone interview only. In-person contact and a detailed psychopathological assessment might have improved the classification of depression and anxiety levels. Additionally, the lack of blinding of outcome assessors represents a potential source of bias; however, the use of standardized and validated questionnaires likely minimized this risk to some extent.

It is important to highlight that we only demonstrate an association between depression and anxiety in a cohort with Long-COVID symptoms. Importantly, we cannot comment on causation. It might well be that symptoms of depression and anxiety are caused by other symptoms of Long-COVID. Furthermore, adherence to the telemedical intervention was not systematically monitored in this study. This was a deliberate decision to replicate a real-world clinical scenario, where strict adherence to telemedical interventions can likewise not be enforced. Future studies should consider integrating adherence tracking mechanisms to inform where adherence to telemedical interventions can be improved.

## 5. Conclusions

In conclusion, our study reveals relevant insights into the prevalence of and risk factors for depression and anxiety as part of Long-COVID 12 months after an index COVID-19 infection in a well-characterized cohort with high data quality. Despite a neutral result, it is the first study to systematically assess the impact of telemedical care on the occurrence of symptoms of depression and anxiety as part of Long-COVID. Telemedical care should hence be limited to the acute COVID-19 infection phase. We confirm female sex and active smoking as risk factors, and we establish pre-existing asthma as a new risk factor for depression and anxiety after COVID-19. Based on our findings, public health strategies could include targeted interventions for female patients, active smokers, and individuals with asthma to address their heightened risk for depression and anxiety. Smoking cessation programs and close monitoring of patients with preconditioned asthma during recovery could help mitigate the long-term psychological burden of Long-COVID.

## Figures and Tables

**Table 1:** Baseline characteristics stratified by outcomes.

	Overall (*n* = 573)	Stratified by depression risk			Stratified by risk for anxiety		
		Positive (*n* = 209)	Negative (*n* = 364)	*p*	Positive (*n* = 232)	Negative (*n* = 341)	*p*
Age (years)	46.8 ± 13.3	45.9 ± 13.3	47.3 ± 13.3	0.24	44.4 ± 12.9	48.4 ± 13.4	<0.001^*∗*^
Female	260 (45.4%)	121 (57.9%)	139 (38.2%)	<0.001^*∗*^	130 (56.0%)	130 (38.1%)	<0.001^*∗*^
BMI (kg/m^2^)	28.3 ± 6.2	28.8 ± 6.6	28.0 ± 5.8	0.13	28.1 ± 6.2	28.4 ± 6.2	0.58
Smoker	164 (28.6%)	72 (34.4%)	92 (25.3%)	0.020^*∗*^	80 (34.5%)	84 (24.6%)	0.011^*∗*^
Number of vaccinations against SARS-CoV2 (IQR)	3 (2;3)	3 (2;3)	3 (3;3)	0.05	2 (3;3)	3 (2;3)	0.21
Preexisting conditions							
Hypertension	273 (47.6%)	87 (41.6%)	186 (51.1%)	0.027^*∗*^	102 (44.0%)	171 (50.1%)	0.14
Hyperlipidemia	127 (22.2%)	42 (20.1%)	85 (23.4%)	0.37	46 (19.8%)	81 (23.8%)	0.27
Diabetes mellitus	66 (11.5%)	20 (9.6%)	46 (12.6%)	0.27	17 (7.3%)	49 (14.4%)	0.011^*∗*^
Heart failure	17 (3.0%)	7 (3.3%)	10 (2.7%)	0.68	10 (4.3%)	7 (2.1%)	0.13
Atrial fibrillation	29 (5.1%)	6 (2.9%)	23 (6.3%)	0.08	5 (2.2%)	24 (7.0%)	0.013^*∗*^
Stroke	12 (2.1%)	5 (2.4%)	7 (1.9%)	0.71	5 (2.2%)	7 (2.1%)	0.94
Cancer	43 (7.5%)	18 (8.6%)	25 (6.9%)	0.45	19 (8.2%)	24 (7.0%)	0.61
COPD	7 (1.2%)	4 (1.9%)	3 (0.8%)	0.27	2 (0.9%)	5 (1.5%)	0.52
Asthma	82 (14.3%)	39 (18.7%)	43 (11.8%)	0.025^*∗*^	47 (20.3%)	35 (10.3%)	<0.001^*∗*^
Autoimmune disease	70 (12.2%)	32 (15.3%)	38 (10.4%)	0.09	40 (17.2%)	30 (8.8%)	0.003^*∗*^
Liver disease	41 (7.2%)	19 (9.1%)	22 (6.0%)	0.171	23 (9.9%)	18 (5.3%)	0.038^*∗*^

*Note*: The table reports baseline characteristics for the overall cohort stratified by depression and anxiety risk. Variables are expressed as absolute and relative frequencies for discrete measures or mean and standard deviation and median with interquartile range if applicable. *p*-Values are calculated by fitting univariable logistic regression models.

*⁣* ^*∗*^ *p*-values <0.05 are considered significant.

**Table 2:** Symptoms stratified by outcomes.

	Overall (*n* = 573)	Stratified by depression risk			Stratified by risk for anxiety		
		Positive (*n* = 209)	Negative (*n* = 364)	*p*	Positive (*n* = 232)	Negative (*n* = 341)	*p*
Symptoms at 12 months	234 (40.8%)	140 (67.0%)	94 (25.8%)	<0.001^*∗*^	137 (59.1%)	97 (28.4%)	<0.001^*∗*^

Severity of symptoms after 12 months	Mild 170 (29.7%)	95 (45.5%)	75 (20.6%)	<0.001^*∗*^	97 (41.8%)	73 (21.4%)	<0.001^*∗*^
	Moderate 50 (8.7%)	36 (17.2%)	14 (3.8%)	<0.001^*∗*^	31 (13.4%)	19 (5.6%)	<0.001^*∗*^
	Severe 14 (2.4%)	9 (4.3%)	5 (1.4%)	0.045^*∗*^	9 (3.9%)	5 (1.5%)	0.10

Disablement due to symptoms	147 (25.7%)	101 (48.3%)	46 (12.6%)	<0.001^*∗*^	97 (41.8%)	50 (14.7%)	<0.001^*∗*^

Aggravation of pre-existing disease	90 (15.7%)	47 (22.5%)	43 (11.8%)	0.001^*∗*^	57 (24.6%)	33 (9.7%)	<0.001^*∗*^

Symptom details							
Skin changes	102 (17.8%)	61 (29.2%)	41 (11.3%)	<0.001^*∗*^	69 (29.7%)	33 (9.7%)	<0.001^*∗*^
Thoracal pain	26 (4.5%)	17 (8.1%)	9 (2.5%)	0.003^*∗*^	19 (8.2%)	7 (2.1%)	<0.001^*∗*^
Cough	47 (8.2%)	24 (11.5%)	23 (6.3%)	0.039^*∗*^	28 (12.1%)	19 (5.6%)	0.008^*∗*^
Dyspnea	77 (13.4%)	52 (24.9%)	25 (6.7%)	<0.001^*∗*^	49 (21.1%)	28 (8.2%)	<0.001^*∗*^
Palpitations	48 (8.4%)	36 (17.2%)	12 (3.3%)	<0.001^*∗*^	35 (15.1%)	13 (3.8%)	<0.001^*∗*^
Impaired concentration	74 (12.9%)	59 (28.2%)	15 (4.1%)	<0.001^*∗*^	60 (25.9%)	14 (4.1%)	<0.001^*∗*^
Impaired sleep	71 (12.4%)	46 (22.0%)	25 (6.9%)	<0.001^*∗*^	48 (20.7%)	23 (6.7%)	<0.001^*∗*^
Headache	62 (10.8%)	46 (22.0%)	16 (4.4%)	<0.001^*∗*^	47 (20.3%)	15 (4.4%)	<0.001^*∗*^
Increased level of pain	24 (4.2%)	19 (9.1%)	5 (1.4%)	<0.001^*∗*^	19 (8.2%)	5 (1.5%)	<0.001^*∗*^
Impaired smell	49 (8.6%)	30 (14.4%)	19 (5.2%)	<0.001^*∗*^	32 (13.8%)	17 (5.0%)	<0.001^*∗*^
Memory problems	62 (10.8%)	46 (22.0%)	16 (4.4%)	<0.001^*∗*^	43 (18.55)	19 (5.6%)	<0.001^*∗*^
Impairment of speech	25 (4.4%)	23 (11.0%)	2 (0.5%)	<0.001^*∗*^	20 (8.6%)	5 (1.5%)	<0.001^*∗*^
Muscular pain	49 (8.6%)	36 (17.2%)	13 (4.6%)	<0.001^*∗*^	38 (16.4%)	11 (3.2%)	<0.001^*∗*^
Sight problems	25 (4.4%)	19 (9.1%)	6 (1.6%)	<0.001^*∗*^	18 (7.8%)	7 (2.1%)	0.001^*∗*^

*Note*: The table reports symptoms for the overall cohort stratified by depression and anxiety risk. Variables are expressed as absolute and relative frequencies for discrete measures or mean and standard deviation and median with interquartile range if applicable. *p*-Values are calculated by Fisher's exact test.

*⁣* ^*∗*^ *p*-values <0.05 are considered significant.

**Table 3:** Symptoms stratified by randomization group.

	Overall (*n* = 573)	Stratified by randomization group		
		Intervention (*n* = 288)	Control (*n* = 285)	*p*
Symptoms at 12 months	234 (40.8%)	116 (40.3%)	118 (41.4%)	0.80

Severity of symptoms 12 months				
Mild	170 (29.7%)	84 (29.2%)	86 (30.2%)	0.86
Moderate	50 (8.7%)	26 (9.0%)	24 (8.4%)	0.88
Severe	14 (2.4%)	6 (2.1%)	8 (2.8%)	0.60
Disablement due to symptoms	147 (25.7%)	77 (26.7%)	70 (24.6%)	0.57
Aggravation of pre-existing disease	90 (15.7%)	51 (17.7%)	39 (13.7%)	0.41
No return to work	46 (8.0%)	20 (6.9%)	26 (9.1%)	0.04^*∗*^
Symptom details				
Skin changes	102 (17.8%)	47 (16.3%)	55 (19.3%)	0.62
Thoracal pain	26 (4.5%)	12 (4.2%)	14 (4.9%)	0.84
Cough	47 (8.2%)	27 (9.4%)	20 (7.0%)	0.56
Dyspnea	77 (13.4%)	35 (12.2%)	42 (14.7%)	0.65
Palpitations	48 (8.4%)	31 (10.8%)	17 (6.0%)	0.12
Impaired concentration	74 (12.9%)	41 (14.2%)	33 (11.6%)	0.62
Impaired sleep	71 (12.4%)	35 (12.2%)	36 (12.6%)	0.91
Headache	62 (10.8%)	33 (11.5%)	29 (10.2%)	0.85
Increased level of pain	24 (4.2%)	12 (4.2%)	12 (4.2%)	1.00
Impaired smell	49 (8.6%)	24 (8.3%)	25 (8.8%)	0.92
Memory problems	62 (10.8%)	30 (10.4%)	32 (11.2%)	0.89
Impairment of speech	25 (4.4%)	15 (5.2%)	10 (3.5%)	0.61
Muscular pain	49 (8.6%)	27 (9.4%)	22 (7.7%)	0.77
Sight problems	25 (4.4%)	11 (3.8%)	14 (4.9%)	0.73

*Note*: The table reports outcomes for the overall cohort with complete 12-month FU stratified by randomization group. Parameters are expressed as absolute and relative frequencies for discrete measures. *p*-Values are calculated by Fisher's exact test.

*⁣* ^*∗*^ *p*-values <0.05 are considered significant.

**Table 4:** Multivariable analysis.

Variable	Depression		Anxiety	
	OR (CI)	*p*	OR (CI)	*p*
Gender at birth: female	2.18 (1.54−3.12)	<0.001^*∗*^	2.01 (1.42−2.84)	<0.001^*∗*^
Smoker: yes	1.63 (1.11−2.38)	0.012^*∗*^	1.72 (1.18−2.50)	0.005^*∗*^
Asthma: yes	1.63 (1.00−2.66)	0.047^*∗*^	2.17 (1.34−3.55)	0.002^*∗*^

*Note*: The table reports odds ratios (OR) with 95% confidence intervals (CIs) for the multivariable analysis of predictors for depression and anxiety.

*⁣* ^*∗*^ *p*-values <0.05 are considered significant.

## Data Availability

The data that support the findings of this study are available on request from the corresponding author. The data are not publicly available due to privacy restrictions.
